# Behavioural and neurobiological consequences of macrophage migration inhibitory factor gene deletion in mice

**DOI:** 10.1186/s12974-015-0387-4

**Published:** 2015-09-04

**Authors:** Cecilie Bay-Richter, Shorena Janelidze, Analise Sauro, Richard Bucala, Jack Lipton, Tomas Deierborg, Lena Brundin

**Affiliations:** Translational Neuropsychiatry Unit, Department of Clinical Medicine, Aarhus University, Risskov, Denmark; Clinical Memory Research Unit, Department of Clinical Sciences, Lund University, Malmö, Sweden; Department of Psychiatry and Behavioral Medicine, Michigan State University, Grand Rapids, MI USA; Department of Medicine, Yale School of Medicine, New Haven, CT USA; Department of Translational Science and Molecular Medicine, Michigan State University, Grand Rapids, MI USA; Experimental Neuroinflammation Laboratory, Department of Experimental Medical Science, BMC, Lund University, Lund, Sweden; Laboratory of Behavioral Medicine, Van Andel Research Institute, Grand Rapids, MI USA

**Keywords:** Macrophage migration inhibitory factor, Depression, Cytokines, Dopamine, Inflammation

## Abstract

**Background:**

Evidence from clinical studies and animal models show that inflammation can lead to the development of depression. Macrophage migration inhibitory factor (MIF) is an important multifunctional cytokine that is synthesized by several cell types in the brain. MIF can increase production of other cytokines, activates cyclooxygenase (COX)-2 and can counter-regulate anti-inflammatory effects of glucocorticoids. Increased plasma levels of MIF are associated with hypothalamic–pituitary–adrenal (HPA) axis dysregulation and depressive symptoms in patients. In contrast, MIF knockout (KO) mice have been found to exhibit increased depressive-like behaviour. The exact role for MIF in depression is therefore still controversial. To further understand the role of MIF in depression, we studied depressive-like behaviour in congenic male and female MIF KO mice and wild-type (WT) littermates and the associated neurobiological mechanisms underlying the behavioural outcome.

**Methods:**

MIF KO and WT mice were tested for spontaneous locomotor activity in the open-field test, anhedonia-like behaviour in the sucrose preference test (SPT), as well as behavioural despair in the forced swim test (FST) and tail suspension test (TST). Brain and serum levels of cytokines, the enzymes COX-2 and indoleamine-2,3-dioxygenase (IDO) and the glucocorticoid hormone corticosterone were measured by RT-qPCR and/or high-sensitivity electrochemiluminescence-based multiplex immunoassays. Monoamines and metabolites were examined using HPLC.

**Results:**

We found that MIF KO mice of both sexes displayed decreased depressive-like behaviour as measured in the FST. In the TST, a similar, but non-significant, trend was also found. IFN-γ levels were decreased, and dopamine metabolism increased in MIF KO mice. Decreased brain IFN-γ levels predicted higher striatal dopamine levels, and high dopamine levels in turn were associated with reduced depressive-like behaviour. In the SPT, there was a sex-specific discrepancy, where male MIF KO mice showed reduced anhedonia-like behaviour whereas female KO mice displayed increased anhedonia-like behaviour. Our results suggest that this relates to the increased corticosterone levels detected in female, but not male, MIF KO mice.

**Conclusions:**

Our findings support that MIF is involved in the generation of depressive-like symptoms, potentially by the effects of IFN-γ on dopamine metabolism. Our data further suggests a sex-specific regulation of the involved mechanisms.

## Introduction

Depression is a severe and debilitating disease and the lifetime prevalence for major depressive disorder has in some populations been reported to be more than 20 % [1]. Current antidepressant medication primarily focuses on modulating the monoamine neurotransmitter systems which only provide sufficient symptom relief to approximately half of the patients [2, 3]. This emphasizes the need for a better understanding of the disease to be able to develop new and improved treatment strategies. Recently, it has been suggested that inflammation may play a key role in the pathophysiology of depression. It has been known for several years that patients without psychiatric history, who receive injections of pro-inflammatory substances (interferons) as treatment for certain forms of cancer, develop depression and suicidality at an increased rate [4–7]. Furthermore, depressive symptoms appear in both humans and animal models when cytokine levels are experimentally elevated by injections of the bacterial endotoxin lipopolysaccharide [8–10]. Interestingly, patients with primary depression (e.g. with no known underlying somatic cause) also have elevated plasma levels of pro-inflammatory cytokines [11–13], and it has even been shown that treatment with anti-inflammatory drugs can alleviate symptoms in depressed patients as measured with the Hamilton Depression Scale [14].

Macrophage migration inhibitory factor (MIF) is an important multifunctional cytokine that is synthesized by several cell types in the brain and is expressed in brain areas associated with the behavioural symptoms of depression [15]. MIF is upregulated in diseases with an inflammatory component, such as cancer, obesity and type 2 diabetes. Further, MIF induces expression of the key pro-inflammatory receptor Toll-like receptor 4 [16, 17], activates cyclooxygenase (COX)-2 [18] and has also been found to counter-regulate the anti-inflammatory effects of glucocorticoids [19–21]. It has also been shown that MIF can increase the cytokine levels of TNF-α, IL-1β and IL-6 [22, 23] which have all been associated with depression. Correspondingly, it has been shown that MIF inhibition leads to downregulation of TNF-α and IFN-γ [24].

A link between MIF and depression has already been suggested but its exact role is still under debate [15]. Clinical studies have found that serum levels of MIF are increased in depressed patients [25] and correlate positively with depressive symptoms as measured with the Beck Depression Inventory short form [26]. Non-responding depressed patients display increased blood levels of MIF mRNA, and antidepressant drugs decrease the blood levels of this cytokine [27]. In line with these clinical findings, an animal model has shown that 4, 8, 12 or 16 weeks of unpredictable chronic mild stress all increases levels of MIF and can lead to depressive-like behaviour [28]. Together, these results indicate that higher levels of MIF are associated with more severe depressive and depressive-like symptoms. Therefore, MIF could potentially constitute a target for future anti-depressive treatments. However, the role of MIF in depression is still controversial as an experimental study showed that MIF KO mice exhibited a higher degree of depressive-like behaviour compared to WT controls as measured using the forced swim test (FST) [29], and another study indicated that the anti-depressive effects of voluntary wheel-running exercise are mediated by *increased* production of MIF [30].

As evident from the above, the effects of MIF and MIF deletion on depressive-like behaviour and the underlying mechanisms involved need to be characterized in detail. In the studies presented here, we aimed to further elucidate the role of MIF in depressive-like behaviour by the use of congenic male and female MIF KO mice and WT littermates. As human depression is a multimodal disease, there is no single test for rodent behaviour that reflects human depression. We assessed the effects of MIF deletion on rodent behaviour utilizing a battery of validated behavioural tests, including the FST, the tail suspension test (TST) and the sucrose preference test (SPT) [31]. The TST and FST are both validated models of behavioural despair in mice. The term “behavioural despair” is used, as it is believed that immobility in this test reflects that the animals have “given up hope of escaping” [32]. Although TST and FST share a common theoretical basis, there are differences between them, and they therefore complement each other. For example, TST avoids problems of hypothermia or motor dysfunction that could interfere with the performance in the swim test, while the FST could overcome the tail climbing problem in the TST [33]. The SPT is a measure of anhedonia-like behaviour; when given a choice of drinking either a sucrose solution or water, rodents will choose to drink the sucrose solution. Animals that do not show this preference are believed to display anhedonia-like behaviour [34]. We selected these behavioural tests to be able to assess both behavioural despair as well as anhedonia-like behaviour.

In addition to testing for depressive-like behaviour, we analysed spontaneous locomotor activity in the open-field test. Spontaneous locomotor activity was examined to determine that the animals did not display motor abnormalities that could be a confounding factor when evaluating the outcome of the FST. Moreover, in order to further understand the underlying neurobiological changes, we analysed the relation of inflammatory factors and monoamine metabolism to the observed behaviour.

## Materials and methods

### Animals

Adult congenic MIF−/− (KO) mice and MIF+/+ (WT) littermates on a C57BL/6 background were used for these experiments. KO and WT mice were age-matched in all experiments and randomised so that both siblings as well as non-siblings were found in each experimental group. The animals were bred and housed at Lund University, Sweden. Genotype was determined by PCR using primers as previously reported [35]. The animals were housed two to six per cage in standard laboratory cages with sawdust bedding, enrichment material and free access to water and food. The holding room had a 12:12 light–dark cycle (light on at 7.00 am). Body weights were monitored throughout the experiment, and all procedures were performed in accordance with national and local laws and were approved by the Regional Animal Ethics Committee in Malmö/Lund, Sweden.

## Behavioural testing

### General procedures

We examined depressive-like behaviour in our animal model by means of the SPT, the FST and the TST. On day 1–8, the animals went through the SPT. On day 4, the habituation for the open field test (OFT) was performed and on day 6, the locomotor activity test. The FST was performed on day 8 and TST 24 h after, on day 9. Number of animals per behavioural group was ≥7 in all groups.

### Open-field test

To ascertain that the animals did not suffer from any gross locomotor disturbances, spontaneous activity in the OFT was assessed. The experiment consisted of a habituation trial of 6 min followed 48 h after by the 6-min test. The OFT was a white Plexiglas box of 45 × 45 cm with 40 cm high walls, and the total distance moved was recorded using the SMART tracking software (Panlab, Harvard apparatus, Spain). Twelve male WT, 10 female WT, 16 male KO and 16 female KO animals went through the OFT.

### Forced swim test

Stillness, a measure of behavioural despair, was examined in the FST for 6 min, using clear Plexiglas cylinders (25 cm high, *ø* 20 cm) which were filled with 25 °C water to a depth where the animals could not touch the bottom with their tails [36]. Stillness was assessed using SMART tracking software (Panlab, Harvard apparatus, Spain). Eight male WT, 7 female WT, 8 male KO and 8 female KO mice went through the FST.

### Tail suspension test

The TST apparatus was a white Plexiglas box with one open side (30 cm × 20 × 20) which in the middle of the ceiling had a hook attached. Adhesive tape was attached to the tail of the mouse (approximately 1 cm away from the tip of the tail), and the mouse was then suspended from the hook in the ceiling, for 10 min [37, 38]. The behaviour of the animal was recorded with a video camera, and stillness was subsequently rated blindly. Eight male WT, 7 female WT, 8 male KO and 8 female KO mice went through the TST, but one animal had to be excluded as it performed tail climbing and therefore correct behaviour could not be evaluated.

### Sucrose preference test

For the SPT, animals were placed in separate cages, with access to two bottles; one containing tap water and the other a 2 % sucrose solution, for 5 h every day and for 8 days during the wake period of the animals’ diurnal cycle. The bottles were counterbalanced to avoid side preferences. Percent sucrose preference was calculated as $\frac{\mathrm{sucrose}\ \mathrm{consumption}\ \left(\mathrm{g}\right) \times 100}{\mathrm{sucrose}\ \mathrm{consumption}\ \left(\mathrm{g}\right) + \mathrm{water}\ \mathrm{consumption}\ \left(\mathrm{g}\right)}$. Twelve male WT, 10 female WT, 16 male KO and 16 female KO animals underwent the SPT.

### Termination and sample preparation

After the last behavioural test, animals were deeply anesthetized with pentobarbital (100 mg/kg) and blood was collected from the heart. Brain regions of interest were immediately isolated on ice, snap-frozen on dry ice and thereafter stored at −80 °C until further analysis.

### Reverse transcription quantitative PCR (RT-qPCR)

RNA from brain tissue (hippocampus and prefrontal cortex (PFC)) was extracted using Trizol reagent (Invitrogen, Sweden) followed by RNeasy mini kit (Qiagen, Germany), according to the manufacturer’s instructions. One microgram of total RNA was reverse transcribed into cDNA using Super-Script III (Invitrogen, Sweden) following the manufacturer’s instructions. RT-qPCR was conducted on a C1000 thermal cycler with CFX 96 real-time system (Bio-Rad, Sweden) using Maxima SYBRgreen qPCR Mix (Fermentas, Sweden) in a 20 μl reaction with Hprt1 and Ppia as the control genes. All samples were run in triplicates. The following mRNA sequences were examined: IL-1β, IL-6, TNF-α, IFN-γ, IDO1 and COX-2. IFN-γ was undetectable using RT-qPCR for a total of ten samples (1 male WT, 3 female WT, 3 male KO, 3 female KO). These samples were assigned a Ct value of 40. Primer sequences are found in Table 1. Data were analysed using the Pfaffl method [39]. Tissue from 8 male WT, 7 female WT, 8 male KO and 8 female KO animals was available for RT-qPCR experiments.

**Table 1:** Primers used for RT-qPCR experiments

	Primer sequences
Hprt1 forward	5′-CTCATGGACTGATTATGGACAGGAC-3′
Hprt1 reverse	5′-GCAGGTCAGCAAAGAACTTATAGCC-3′
Ppia forward	5′-TATCTGCACTGCCAAGACTGAATG-3′
Ppia reverse	5′-CTTCTTGCTGGTCTTGCCATTCC-3′
IFN-γ forward	5′-CACTGCATCTTGGCTTTGCA-3′
IFN-γ reverse	5′-GCTGATGGCCTGATTGTCTTTC-3′
COX-2 forward	5′-TGCCTGGTCTGATGATGTATGCCA-3′
COX-2 reverse	5′-GTATGTCGCACACTCTGTTGTGCT-3′
IL-1β forward	5′-CAACCAACAAGTGATATTCTCCAT-3′
IL-1β reverse	5′-ATCCACACTCTCCAGCTGCA-3′
IL-6 forward	5′-GAGGATACCACTCCCAACAGACC-3′
IL-6 reverse	5′-AAGTGCATCATCGTTGTTCATACA-3′
TNF-α forward	5′-CATCTTCTCAAAATTCGAGTGACAA-3′
TNF-α reverse	5′-TGGGAGTAGACAAGGTACAACCC-3′
IDO1 forward	5′-TATTGCTGTTCCCTACTG-3′
IDO1 reverse	5′-GGTCTTGACGCTCTACT-3′

### Detection of cytokines

Cytokines were quantified in blood serum using high-sensitivity electrochemiluminescence-based multiplex immunoassay (MesoScale Discovery, USA). We used the ultrasensitive pro-inflammatory cytokine multiplex kit to measure IL-6, IL-1β, KC/GRO, IFN-γ, IL-10, IL12p70 and TNF-α (Meso Scale Discovery, Rockville, USA). Fifty-microliter reactions were carried out, and the multiplex plates were analysed on a SECTOR 6000 instrument following the manufacturer’s instructions. All samples were run in duplicates. Serum from 12 male WT, 10 female WT, 16 male KO and 16 female KO was available for the multiplex immunoassay. For one assay, IL-1β was not detectable due to technical issues and therefore, IL-1β was detected in serum from only 9 male WT, 3 female WT, 8 male KO and 4 female KO animals.

### High-performance liquid chromatography

Monoamine neurotransmitters and their metabolites were examined in the striatum of the animals using high-performance liquid chromatography (HPLC) as described previously [40]. These included dopamine (DA) and its metabolites 3,4-dihydroxyphenylacetic acid (DOPAC) and homovanillic acid (HVA), serotonin (5-HT) and its metabolite 5-hydroxyindoleacetic acid (5-HIAA) and norepinephrine (NE) and its metabolite 3-methoxy-4-hydroxyphenylglycol (MHPG). Striatal tissue from 12 male WT, 8 female WT, 16 male KO and 15 female KO animals was available for the HPLC analyses.

### Corticosterone assay

Corticosterone was quantified using a commercially available assay (DetectX, Arbor Assays, USA). Fifty microliters of blood serum was used, and all samples were run in duplicates. The assay was performed according to the manufacturer’s instructions. Serum was available from 8 male WT, 6 female WT, 10 male KO and 7 female KO animals for the corticosterone assay.

### Statistical analysis

Statistical analysis was performed using SPSS 19 software. For all analysis, 2 × 2 univariate ANOVAs were performed and when appropriate, i.e. when a significant interaction between genotype and sex was found, this was followed by planned comparisons Student’s independent *t*-tests comparing genotype within each sex. Non-normally distributed data were ln transformed. Linear regression models were used to investigate the impact of the concentration of biological analytes on behavioural outcomes. Correlation analysis was performed using Pearson’s *r*. Alpha level of significance was set at *p* < 0.05.

## Results

### Effects of MIF deletion on behaviour

#### MIF KO mice show decreased behavioural despair

**Fig. 1:**
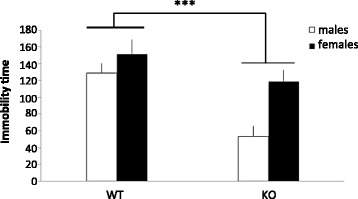
Immobility time (s/6 min) in the forced swim test. *Columns* represent mean + SEM. ***p < 0.001 compared to WT animals (ANOVA) (*n* = 7–8 per group)

In the FST, KO animals of both sexes were significantly less immobile than WT littermates, indicating a lower level of depressive-like behaviour in the KO mice (Fig. 1). There was an effect of genotype (*F*(1,27) = 14.784, *p* = 0.001) and sex (*F*(1,27) = 9.822, *p* < 0.01) with males being less immobile than females independent of genotype (genotype × sex interaction (*F*(1,27) = 2.374, NS)).

**Fig. 2:**
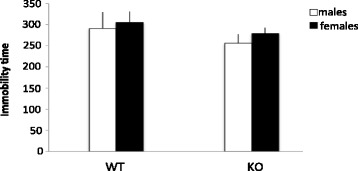
Immobility time (s/10 min) in the tail suspension test. *Columns* represent mean + SEM. (*n* = 7–8 per group)

In the TST, a non-significant trend towards a genotype effect was also found (*F*(1,26) = 3.946, *p* = 0.058), with KO animals showing less immobility compared to WT controls, which is in line with the FST results above (Fig. 2).

#### Increased sucrose preference in male MIF KO mice

**Fig. 3:**
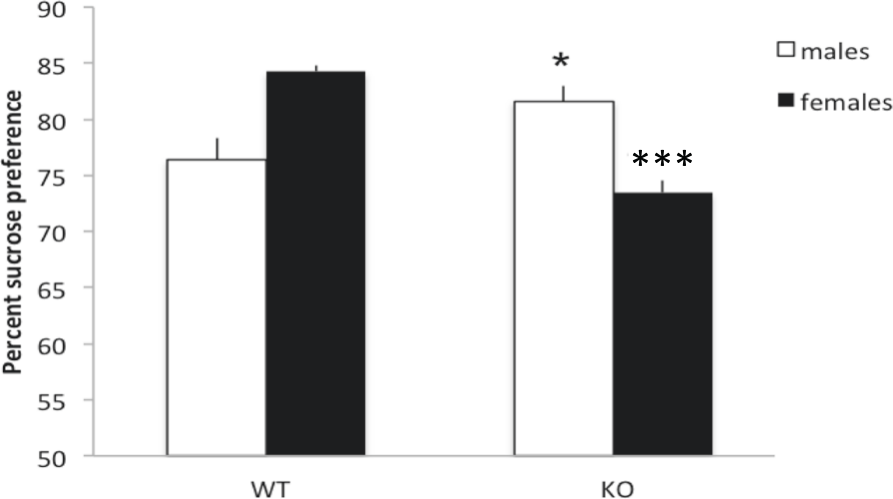
Percent sucrose preference in the sucrose preference test. *Columns* represent mean + SEM. *p = 0.05, ***p < 0.001 compared to the WT group of same sex in post hoc *t* tests (*n* = 10–16 animals per group)

In the SPT, we found an effect of genotype (*F*(1,50) = 4.295, *p* < 0.05) and a genotype × sex interaction (*F*(1,50) = 35.731, *p* < 0.001). Post hoc comparisons showed that male MIF KO mice had an increased preference for sucrose compared to WT animals, indicating that they also in this test demonstrated a lower degree of depressive-like behaviour. In contrast, female MIF KO mice showed less preference for sucrose than the WT females (see Fig. 3).

#### MIF KO mice display slight hypolocomotion in the open-field test

**Fig. 4:**
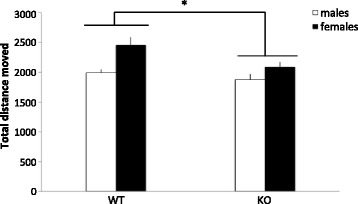
Spontaneous locomotor activity in the open field (cm/6 min) in MIF KO compared to WT littermate controls. *Columns* represent mean + SEM. *p < 0.05 compared to WT animals (ANOVA) (*n* = 10–16 animals per group)

Locomotor activity in the OFT was performed to evaluate motor performance of the MIF KO mice compared with WT littermates, which is important for the FST interpretation. An effect of genotype (*F*(1,50) = 6.143, *p* < 0.05) and sex (*F*(1,50) = 11.667, *p* = 0.001) but no genotype × sex interaction (*F*(1,50) = 1.606, NS) was found. As can be seen in Fig. 4, MIF KO animals showed a slightly decreased locomotor activity in the OFT compared to WT. Despite the lack of a significant genotype × sex interaction, the decrease in locomotor activity appears primarily in female animals. The decreased spontaneous locomotor activity in the OFT did not confound the performance in the FST, as MIF KO animals were found to move significantly *more* in the FST test than WT controls.

#### Food intake is unaltered in MIF KO mice

To examine whether caloric intake differed between the experimental groups, food intake was also measured. No difference was found (all *F* values <0.03). In addition, bodyweights did not differ between genotype (all *F* values <2).

### MIF deletion causes sex-specific alteration of IFN-γ mRNA expression

**Fig. 5:**
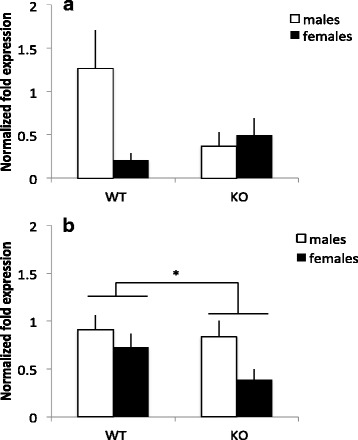
Normalized fold mRNA expression of IFN-γ in MIF KO and WT mice in the **a** prefrontal cortex and **b** hippocampus. *Columns* represent mean + SEM. *p < 0.05 compared to WT animals (ANOVA) (for **(a)**, *n* = 7–8 per group, and for **(b)**, *n* = 6–8 per group)

Reverse transcriptase qPCR on prefrontal cortex (PFC tissue) showed an altered IFN-γ mRNA expression in KO mice compared to WT mice and that this expression differed between the sexes. This was found as the 2 × 2 univariate ANOVA revealed an interaction between genotype and sex for IFN-γ mRNA expression in the PFC (*F*(1,25) = 4.457, *p* < 0.05). This suggests that the alteration of IFN-γ expression caused by MIF deletion is sex specific (Fig. 5). Post hoc comparisons were non-significant, but the tendency is towards a decreased mRNA expression of IFN-γ in male KO mice and possibly an increased IFN-γ expression in female KO mice.

In the hippocampus, the mRNA expression of IFN-γ was also altered; here, KO mice of both sexes displayed a lower mRNA expression than WT animals. This was revealed in the 2 × 2 univariate ANOVA, where an effect of genotype (*F*(1,27) = 4.346, *p* < 0.05) as well as sex (*F*(1,27) = 5.383, *p* < 0.05) was found. MIF KO animals had lower expression of IFN-γ in the hippocampus, and the females had lower expression than in males (Fig. 5b). The expression of mRNA for all other cytokines examined, COX-2 or IDO (see Table 1) were not altered in MIF KO compared to WT animals (all *F*’s <1.0).

**Fig. 6:**
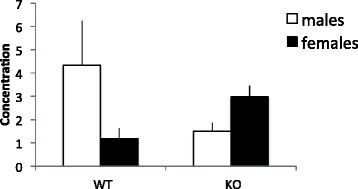
Blood serum expression (pg/ml) of IFN-γ. *Columns* represent mean + SEM

Similar changes were found in the peripheral blood serum for IFN-γ. Here, as seen in the PFC, IFN-γ levels were found altered in KO mice compared to WT mice and this effect differed between sexes. This was found in the 2 × 2 univariate ANOVA where a significant genotype × sex interaction was revealed (*F*(1,16) = 4.788, *p* < 0.05). As can be seen in Fig. 6, MIF KO males appear to display lower levels of IFN-γ than their WT littermates. Interestingly, the female MIF KO mice displayed higher levels of IFN-γ in the blood compared to their WT counterparts. No significant genotype effects were found for other serum cytokines (all *F*’s <3.5).

### The effect of MIF deletion on monoamine metabolite levels

**Fig. 7:**
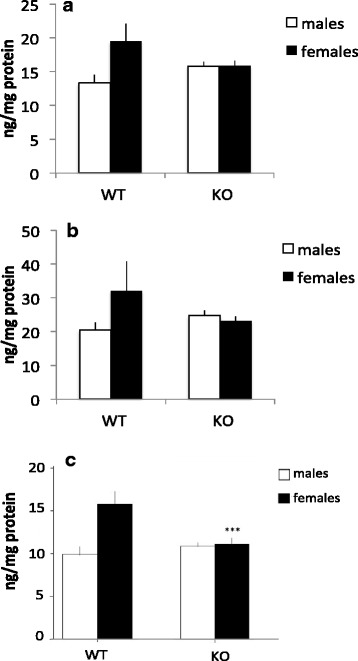
Monoamine metabolite levels in the striatum from WT and MIF KO animals. **a** HVA, **b** DOPAC and **c** 5-HIAA expression. *Columns* represent mean + SEM. ***p < 0.001 compared to the WT group of same sex in post hoc *t* tests (*n* = 8–16 animals per group)

Levels of the dopamine metabolite HVA differed between WT and KO animals, and this difference was dependent of sex; male KO mice displayed slightly increased levels of HVA whereas female KO mice displayed decreased levels of HVA. The 2 × 2 univariate ANOVA showed that a significant genotype × sex interaction (*F*(1,47) = 6.418, *p* = 0.01) was present as well as a sex effect (*F*(1,47) = 6.609, *p* = 0.01), with females, independent of genotype, displaying higher levels of HVA. No overall genotype effect was found (*F* < 0.3). For DOPAC, the pattern was similar; DOPAC differed between WT and KO animals and this difference was dependent of sex; male KO mice displayed slightly increased levels of DOPAC whereas female KO mice displayed decreased levels of DOPAC as evident by the significant genotype × sex interaction (*F*(1,47) = 4.171, *p* < 0.05). No overall genotype or sex effects (*F*’s <2.5) were found (Fig. 7a, b). No significant differences between groups were found for DA (all *F*’s <2.0). In summary, we find sex-specific changes for HVA and DOPAC but not for DA.

The levels of the serotonin metabolite 5-HIAA were altered in MIF KO mice. Overall, MIF KO mice displayed reduced levels of this metabolite, this effect primarily being driven by a marked decrease in the female KO animals, suggesting that MIF deletion in female mice have a specific effect on serotonin metabolites. A significant genotype (*F*(1,47) = 10.900, *p* < 0.01), sex (*F*(1,47) = 18.720, *p* < 0.001) and a genotype × sex interaction (*F*(1,47) = 12.582, *p* < 0.001) was found. MIF KO females showed a statistically significant decrease in 5-HIAA compared to WT females (Fig. 7c).

#### Association between monoamines, inflammation and behaviour

**Fig. 8:**
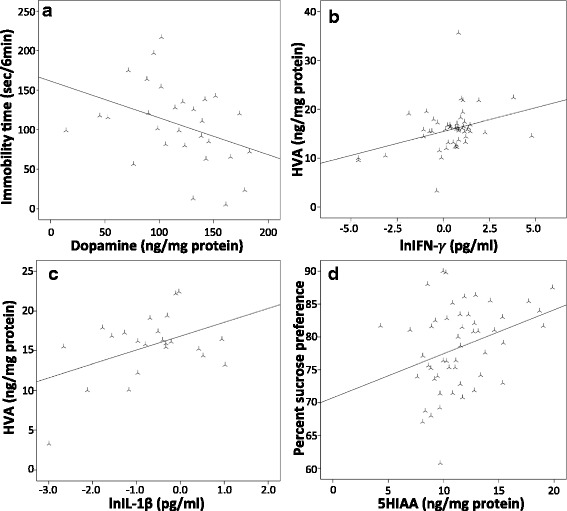
**a** Immobility in FST versus striatal DA levels, **b** correlation between serum IFN-γ and striatal HVA levels, **c** correlation between serum IL-1β and striatal HVA levels and **d** correlation between striatal 5-HIAA and sucrose preference

DA levels correlated negatively with immobility in the FST in all animals (*r* = −0.380, *p* < 0.05) indicating that higher levels of DA are associated with the reduced depressive-like behaviour observed in the MIF KO mice (see Fig. 8a).

The inflammatory cytokines IFN-γ and IL-1β levels correlated positively with HVA levels (*r* = 0.346, *p* = 0.01; *r* = 0.446, *p* < 0.05) showing a relationship between the degree of inflammation and dopamine metabolism (Fig. 8b, c). In a linear regression model including sex and genotype, we found that the serum concentration of IFN-γ was a significant predictor of striatal DA levels (*β* = 0.148; *p* < 0.05), where high levels of IFN-γ corresponded to low DA levels in the striatum.

We also found a positive relationship between sucrose preference and 5-HIAA in all animals (*r* = 0.328, *p* = 0.01) (Fig. 8d). Female MIF KO mice displayed low 5-HIAA levels and also exhibited decreased sucrose preference, as mentioned previously. The reduced 5-HIAA could thus possibly represent a biological mechanism involved in this behavioural change observed in the female MIF KO mice (see Figs. 3 and 7c).

### The effect of MIF deletion on corticosterone levels

**Fig. 9:**
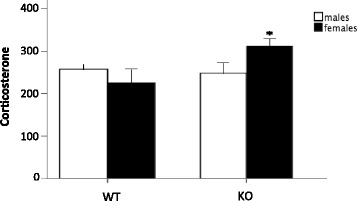
Corticosterone concentration (ng/mL) in MIF WT and KO animals. *Columns* represent mean + SEM. *p < 0.05 compared to WT equivalents (*n* = 7–14 animals per group)

For blood serum levels of corticosterone, a significant genotype × sex interaction was found (*F*(1,35) = 4.326, *p* < 0.05). Female MIF KO mice had significantly elevated corticosterone levels compared to their WT counterparts (Fig. 9).

## Discussion

In summary, we show that deletion of the MIF gene led to reduced behavioural despair in mice of both sexes, as assessed in the FST and with a similar trend in the TST. The IFN-γ mRNA levels were reduced in the hippocampus of the MIF KO mice, and sex-specific alterations of IFN-γ in the PFC and blood serum were found. Moreover, we observed significant changes in the monoamine- and monoamine metabolite levels in the MIF KO mice. Importantly, *lower* serum IFN-γ levels predicted *higher* DA levels, and the DA levels were directly associated with the reduced behavioural despair in the FST in all animals. The results we observed in the TST were very similar to the results in the FST, although they were only trending towards significance. Thus, in this study we have detected a reduced behavioural despair by deleting the MIF gene. The effect was mediated by decreased levels of IFN-γ, which predicted higher levels of brain dopamine.

Our finding that the MIF KO mice displayed reduced depressive-like behaviour in the FST is paralleled by recent clinical findings. Elevated plasma MIF levels have been shown to be associated with higher depression scores in otherwise healthy subjects as measured by the Beck Depression Inventory short form [26], and in pregnant women, higher depression scores assessed using the Center for Epidemiologic Studies Depression Scale were also associated with elevated MIF levels after influenza vaccination [41]. Furthermore, patients suffering from major depressive disorder (MDD) had elevated levels of MIF compared to healthy controls [25]. Recently, Cattaneo and colleagues studied a group of patients suffering from MDD and measured mRNA levels of several cytokines in serum leukocytes. They found increased MIF mRNA levels at baseline in patients, and moreover that MIF levels decreased more than other measured cytokines during treatment [42]. Although ample clinical data support a role of MIF in depression, one study failed to find any connection between blood serum levels of MIF and depressive symptoms assessed by the Zung Self-Rating Depression Scale in a cohort of 209 healthy medical students [43].

This current study is the first time that sex-specific effects of MIF deletion have been assessed at the behavioural and neurobiological level. Interestingly, we found a sex-specific effect of the MIF KO in the sucrose preference test. Male MIF KO animals showed increased sucrose preference, whereas females showed decreased sucrose preference compared to the WT littermates. This behavioural discrepancy between the sexes was paralleled by an increase in plasma corticosteroids and lower brain levels of 5-HIAA in the female mice than their WT counterparts. Clinical depression can be stress-related and increased levels of glucocorticoid hormones such as cortisol have been closely related to the pathogenesis of depression [44, 45]. Chronically elevated cortisol levels and hypothalamic–pituitary–adrenal (HPA) axis hyperactivity is associated with reduced hippocampal size as well as reduced brain 5-HT synthesis and low CSF 5-HIAA levels [46]. Such changes are believed to be associated with depression. Further, it has been suggested that females are more likely to experience an imbalance in plasma levels of cortisol due to hormonal changes during the menstrual cycle [47]. The rodent equivalent of cortisol is corticosterone. MIF is known to counter-regulate glucocorticoid action that reverses glucocorticoid-induced immunosuppression and, in particular, glucocorticoid-induced pro-inflammatory cytokine inhibition [48]. Our present results show that female MIF KO mice displayed significantly increased corticosterone levels. Such increase could underlie some of the observed sex differences caused by the MIF deletion, including the sex-specific decrease in 5-HIAA seen in female KO mice. Further, we see a positive correlation between 5-HIAA levels and sucrose preference. The increased corticosterone levels and subsequent decrease in 5-HIAA could therefore be the underlying factor for the sex difference seen in the SPT. We here studied the effect of MIF deletion on circulating corticosterone levels. Female MIF KO mice displayed significantly increased corticosterone levels, indicating that HPA axis activation could underlie some of the observed gender differences caused by the MIF deletion.

The decreased sucrose preference in female mice is in line with findings in MIF-KO mice by Conboy and colleagues [29]. In addition to a decreased sucrose preference, the study by Conboy and colleagues also reports increased immobility in the FST [29], which is in contrast to our findings in the current report. A difference between these two studies is that in our present study, WT littermates serve as controls whereas in the Conboy et al. [29] study, control animals were obtained from a commercial breeding facility. Importantly, the sex of the MIF KO mice was not reported in the Conboy study, so to our knowledge, our current study is the first to examine the behavioural effects of MIF deletion in both sexes. It is worth mentioning that the baseline stress between litters of mice, experimental time point and different laboratories can also affect the outcome of animal behaviour in general, and particularly the outcome of depressive-like behaviour [49]. Further, a limitation with this and other studies using constitutive KO animals is that compensatory mechanisms such as up- or downregulation of genes cannot be completely excluded. To further elucidate this, experiments using conditional MIF KO mice should be performed.

Hitherto, most experiments have been performed solely in male animals although recent guidelines from, e.g. NIH, now encourage the use of both sexes in pre-clinical experiments [50]. The results presented here stress the importance of an approach using both male and female animals, as distinct sex differences were found. The sex-based differences observed in this study are moreover in line with recent reports showing that female MIF KO mice have larger infarcts and increased microglial activity than WT females and male MIF KO mice after experimental stroke [51]. An important difference between male and female mice was the increased levels of corticosterone in female MIF KO mice, indicating that these animals have a dysregulated HPA axis. Indeed, corticosterone is known downregulate the expression of the cell survival gene Bcl-2 that is important for stroke outcome [52].

We found evidence of reduced IFN-γ expression both in the brain and in the blood of MIF KO animals. Supporting these results, it has been shown that MIF inhibition downregulates the in vivo secretion of IFN-γ measured in spleen mononuclear cells, harvested from mice following 14 days of treatment with a MIF inhibitor [24]. IFN-γ could be an important link between MIF and depressive-like behaviour, as it is known that IFN-γ administration can induce depressive symptoms in humans and depressive-like behaviour in animals, possibly through the activation of the enzyme IDO and thereby the kynurenine pathway which is thought to be related to depression [53]. Patients treated with IFN as a therapy against hepatitis and certain forms of cancer develop depression and suicidality at a significantly increased rate [5, 54]. Moreover, postmortem studies of depressed patients showed increased IFN-γ expression in the Brodmann Area 10 (anterior PFC) [55]. Psychiatric symptoms have been shown to be associated with an IFN-γ gene (+874) T/A gene polymorphism, as hepatitis C patients treated with interferons were more likely to experience depressive symptoms when they had the T (high producer) allele of IFN-γ (+874) gene than patients with the A (low producer) allele [56]. In an animal model, BCG-induced depressive-like behaviour was completely abolished in IFN-γ KO mice [57]. Therefore, IFN-γ might be a key player in the development of depressive-like behaviour. Interestingly, our results indicate a specific role of IFN-γ connected to the MIF deletion, as the other measured cytokines did not differ between WT and KO animals.

We found that the positive effects on depressive-like behaviour in the MIF KO mice were predicted by higher levels of dopamine. In other words, the striatal dopamine content was positively associated with the reduced behavioural despair in the FST. Interestingly, a role of IFN-γ in regulating dopamine levels and dopamine metabolism has previously been reported. IFN-γ indirectly reduces the biosynthesis of dopamine by impairing the oxidation-labile 5,6,7,8-tetrahydrobiopterin, which is rate-limiting for the biosynthesis of dopamine [58]. It is therefore possible that decreased levels of IFN-γ, as seen in the male MIF KO mice, led to a decreased metabolism of dopamine in the male MIF KO mice and consequently higher dopamine levels in these mice. Reduced dopamine levels in the midbrain are associated with depressive-like behaviour, for example, as seen by optogenetic stimulation and inhibition of VTA dopamine neurons [59–61]. In other words, altered expression of IFN-γ could not only lead to both an activation of IDO and the kynurenine pathway implicated in depression, but also to significant changes in the brain dopamine content. We therefore suggest that the observed changes in IFN-γ expression could be a key mechanism responsible for the effects of MIF deletion on behaviour.

## Conclusions

Several reports suggest that MIF is implicated in depression, but its exact biological contribution to the disease is under debate [15]. In this study, we have shed light on possible biological mechanisms by which MIF deletion could impact depressive-like behaviour in rodents. In summary, we found that mice with a genetic deletion of the MIF gene displayed reduced depressive-like behaviour in the FST. Our results suggest that in the absence of MIF, IFN-γ expression is reduced, which in turn affects the biosynthesis of dopamine, leading to increased levels of dopamine metabolites. The dopamine levels were associated with the behavioural changes. For the first time, we also demonstrate sex-specific effects of the MIF deletion, as female MIF KO mice displayed decreased sucrose preference. The behavioural changes in the female MIF KO mice were paralleled by increased corticosteroids and decreased brain 5-HIAA. Thus, our results suggest that MIF has a role in promoting depressive-like behaviours in rodents, and further stresses the importance of examination of sex differences in animal models of depression.
